# Direct Monitoring of Membrane Fatty Acid Changes and Effects on the Isoleucine/Valine Pathways in an *ndgR* Deletion Mutant of *Streptomyces coelicolor*

**DOI:** 10.4014/jmb.2301.01016

**Published:** 2023-03-10

**Authors:** Tae-Rim Choi, Suk Jin Oh, Jeong Hyeon Hwang, Hyun Jin Kim, Nara Shin, Jeonghee Yun, Sang-Ho Lee, Shashi Kant Bhatia, Yung-Hun Yang

**Affiliations:** 1 Department of Microbial Engineering, College of Engineering, Konkuk University, Seoul 05029, Republic of Korea; 2 Department of Forest Products and Biotechnology, Kookmin University, Seoul 02707, Republic of Korea; 3 Department of Pharmacy, College of Pharmacy, Jeju National University, Jeju-si 63243, Republic of Korea; 4 Institute for Ubiquitous Information Technology and Applications, Konkuk University, Seoul 05029, Republic of Korea

**Keywords:** *Streptomyces coelicolor* A3(2), *ndgR*, phospholipid fatty acid, gas chromatography-mass spectrometry

## Abstract

NdgR, a global regulator in soil-dwelling and antibiotic-producing *Streptomyces*, is known to regulate branched-chain amino acid metabolism by binding to the upstream region of synthetic genes. However, its numerous and complex roles are not yet fully understood. To more fully reveal the function of NdgR, phospholipid fatty acid (PLFA) analysis with gas chromatography-mass spectrometry (GC-MS) was used to assess the effects of an *ndgR* deletion mutant of *Streptomyces coelicolor*. The deletion of *ndgR* was found to decrease the levels of isoleucine- and leucine-related fatty acids but increase those of valine-related fatty acids. Furthermore, the defects in leucine and isoleucine metabolism caused by the deletion impaired the growth of *Streptomyces* at low temperatures. Supplementation of leucine and isoleucine, however, could complement this defect under cold shock condition. NdgR was thus shown to be involved in the control of branched-chain amino acids and consequently affected the membrane fatty acid composition in *Streptomyces*. While isoleucine and valine could be synthesized by the same enzymes (IlvB/N, IlvC, IlvD, and IlvE), *ndgR* deletion did not affect them in the same way. This suggests that NdgR is involved in the upper isoleucine and valine pathways, or that its control over them differs in some respect.

## Introduction

*Streptomyces coelicolor* is a soil-dwelling bacterium that produces spores and several secondary metabolites, such as undecylprodigiosin (Red), actinorhodin (Act), methylenomycin, and calcium-dependent antibiotics [1-3]. Its secondary metabolism is initiated by a complex regulatory system, which has been the focus of numerous studies [4-7]. Consequently, NdgR has been characterized as a regulator of amino acid metabolism, quorum sensing, morphological changes, and antibiotic production [8-10]. Along with its orthologs, *NdgR* is located adjacent to *leuCD*, which encodes isopropylmalate dehydratase, and has been identified as a transcriptional regulator that positively controls *leuCD* expression in *S. clavuligerus* and *S. coelicolor* [11, 12]. NdgR is well conserved throughout *Streptomyces* species and many other bacteria, such as *Mycobacteria* and *Corynebacteria* [9, 13].

In addition to regulating neighboring genes, NdgR also regulates the last step of methionine production and controls eight genes in the biosynthetic pathways of branched-chain amino acids (BCAAs), which are at different locations on the genome [11]. Furthermore, NdgR is known to bind directly to the upstream region of three transcription units, including seven genes (*cysA*, *cysM*, *cysN*, *cysD*, *cysC*, *cysH*, and *cysI*) that are involved in sulfur-assimilation metabolism. NdgR also plays a role in the biosynthesis of sulfur-containing thiamine by controlling thiamine monophosphate kinase (ThiL), sulfurylase (MoeB), and phosphomethylpyrimidine kinase (ThiD), which are related to methionine and cysteine [14].

Since NdgR is involved in the production of various primary and secondary metabolites as a global regulator, the *ndgR* deletion mutant expectedly had slower cell growth, defects in differentiation, increased vulnerability to physical shock, and enhanced production of actinorhodin (Act) [8, 14, 15]. Among the various amino acids, the BCAA pathways have been investigated and the direct binding of NdgR was found for several promoters; however, the more direct consequences of BCAA biosynthesis changed due to the *ndgR* deletion having not been well characterized, although it was expected that the decrease in BCAA would result in slower growth, changes in antibiotic production, and vulnerability to the external environment [16-18].

As a major component of the microbial cellular membrane, phospholipid fatty acids (PLFAs) can be analyzed to determine the microbial community composition and monitor dynamic changes in membrane properties [19, 20]. Unlike total lipid analysis, PLFA analysis involves laborious fractionation steps and requires careful treatment of the highly unstable phospholipids [19, 21, 22]. However, PLFA analysis provides more sensitive and direct information on the identity of the membrane phospholipids, as total lipid analysis was known to make identification of some fatty acids difficult [23, 24]. Moreover, as many PLFAs were synthesized from BCAAs [25-27], PLFA analysis can also enable sensitive monitoring of the impact of BCAAs by revealing the changes in membrane fatty acid composition. The membrane is the essential first line of defense from the outer environment for both gram-negative and gram-positive bacteria [28, 29]. Thus, PLFA analysis is a general method applicable to a variety of species [30]; however there are few reports of it having been applied to monitor the membrane changes in *Streptomyces* species.

**Fig. 1:**
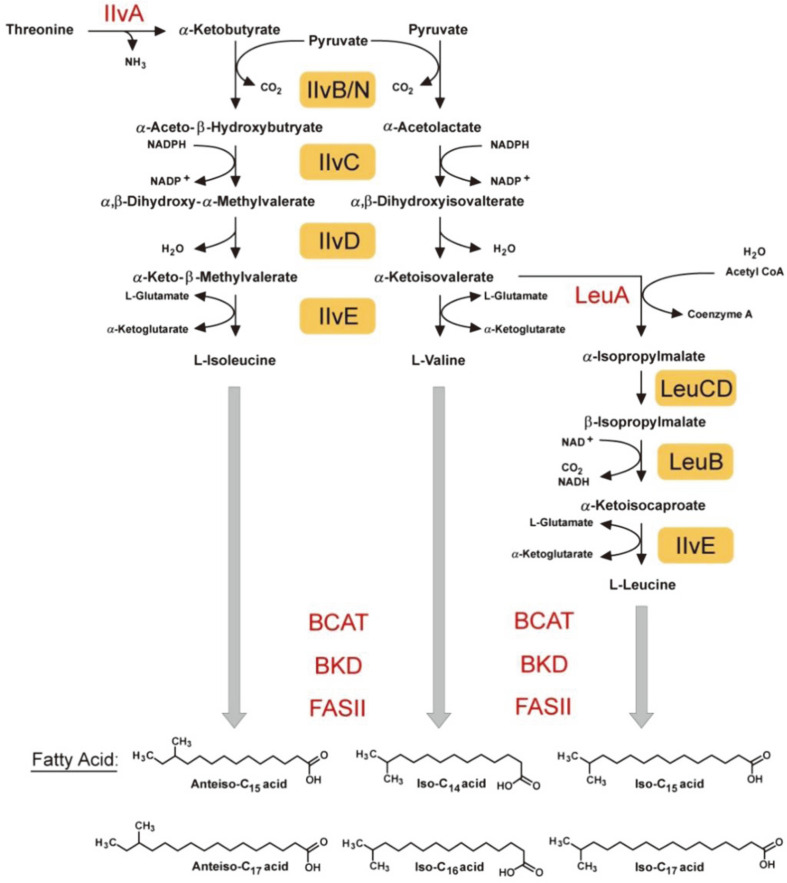
Pathway from branched-chain amino acids (isoleucine, valine and leucine) to branched-chain fatty acids related to membrane phospholipid and involved enzymes. Among the enzymes, bright yellow-colored enzymes are regulated by NdgR.

Thus, considering the direct link of leucine to 13-methylhexadecanoic acid (iso-C15) and 15-methylhexadecanoic acid (iso-C17), isoleucine to 12-methyltetradecanoic acid (anteiso-C15) and 14-methylhexadecanoic acid (anteiso-C17), valine to 12-methyltridecanoic acid (iso-C14) and 14-methylpentadecanoic acid (iso-C16) (Fig. 1), we aimed to elucidate the direct impact on the changes in BCAAs triggered by the *ndgR* deletion in *S. coelicolor*. Using PLFA analysis with GC-MS, we directly monitored membrane changes and the results may provide direct evidence for NdgR regulation of BCAAs and the effects on the membrane composition of *S. coelicolor*.

## Materials and Methods

### Growth of *Streptomyces*

*Streptomyces coelicolor* A3(2) was cultivated in R5 medium consisting of 103 g/l sucrose, 0.25 g/l K2SO4, 10.12 g/l MgCl2·6H2O, 10 g/l glucose, 0.1 g/l casamino acid, 5 g/l yeast extract, 5 mg/l KH2PO4, 20 mM CaCl2, 3 g/l L-proline, 5.72 g/l TES buffer, and 2 ml/l trace element solution (comprising 40 mg/l ZnCl2, 200 mg/l FeCl3·6H2O, 10 mg/l CuCl2·2H2O, 10 mg/l MnCl2·4H2O, 10 mg/l Na2B4O7·10H2O, and 10 mg/l (NH4)6Mo7O24·4H2O). For the plate cultures, 25 g/l agar was added to the same composition of media [8]. The cells were cultivated in 14 ml test tubes containing autoclaved glass beads on a rotary shaker at 200 rpm and 30°C. [15].

### Preparation of PLFA

*Streptomyces coelicolor* A3(2) was spread on R5 agar plates, and cultured for 48–120 h at 20°C or 30°C. At the end of the culture period, the biomass was scraped from the surface and transferred to a vial for lyophilization. Following that, the lipid extraction process was performed in accordance with previous research [31]. Briefly, 1 ml of chloroform and 1 ml of methanol were added to the sample. Thereafter, the mixture was orbital agitated for 2 h at 25°C for lipid extraction. After the addition of 2 ml of distilled water to the mixture and vortexing, followed by centrifugation at 1,500 ×*g* for 5 min, 2 ml of the liquid phase was transferred to glass vials and the sample was evaporated with N2 gas and re-treated with 1 ml of chloroform for suspension. The sample containing the total lipid extract was subjected to column chromatography using silicic acid that binds to lipids [32]. Each lipid in the sample was then eluted using a different solvent; neutral lipids were eluted with chloroform, glycolipids were eluted with acetone, and phospholipids were eluted with methanol. Only methanol-dissolving phospholipids were collected in the glass vial and evaporated with N2 gas. The final analytic samples were prepared with 1 ml of chloroform suspension [22].

### GC-MS Analysis

Prepared PLFA samples were analyzed using a gas chromatography-mass spectrometry (GC-MS) system (USA) equipped with a capillary column (Elite-5 ms, 30 m, 0.25 mm, i.d. 0.25-μm film) as described previously [33]. The GC oven temperature was controlled according to a linear temperature gradient for full resolution of the fatty acids (120°C held for 5 min, increased by 6°C/min to 200°C, increased by 2°C/min to 220°C, and then increased by 10°C/min to 300°C). The injection volume was 1 μl and the injector port temperature was set to 210°C. Mass spectra were obtained by electron ionization at 70 eV, and scan spectra were retained within the range of 45–400 m/z. The selected ion mode was used for the detection and fragmentation analysis of the fatty acids [34].

### Cold Shock Experiment

To confirm the cold shock and amino acid supplement effect hypothesis, *Streptomyces* strains were spread on minimal media with and without amino acids. Minimal media composition was 0.5 g/l K2HPO4, 0.2 g/l MgSO4·7H2O, 0.01 g/l FeSO4·7H2O, 10 g/l *N*-acetylglucosamine, 0.5 g/l L-asparagine, and 25 g/l agar, and 2 g/l amino acid (leucine, isoleucine, and valine) was supplemented accordingly. Plates were cultured at 20°C and 30°C. Cell growth was monitored every 24 h for 120 h. After cultivation, samples were prepared and analyzed as described in the preparation for the PLFA and GC-MS analysis section.

## Results

### PLFA Analysis of Δ*leuCD* and Δ*ndgR* Mutants

BCAAs were the precursors to PLFAs [27, 35], and consequently, changes to each different BCAA would affect the resulting PLFA composition (Fig. 1). Normally, BCAAs such as isoleucine, valine, and leucine were modified to α-keto acids, such as α-keto-β-methylvalerate, α-ketoisovalerate, and α-ketoisocaproate by branched amino acid transaminase. The α-keto acids were then changed to CoA esters, such as 2-methylbutyryl-CoA, isobutyryl-CoA, and isovaleryl-CoA by branched α-keto dehydrogenase (BKD). Finally, fatty acids were produced using the CoA esters by fatty acid synthase (FasII), and they differed in their final chain lengths, having odd numbers of carbons (15, 17, etc.) or even numbers (14, 16, etc.). As changes to these BCAAs affect the PLFA composition, the changes in BCAA synthesis could be monitored in various mutants by analyzing the PLFAs in the membrane using GC-MS.

**Fig. 2:**
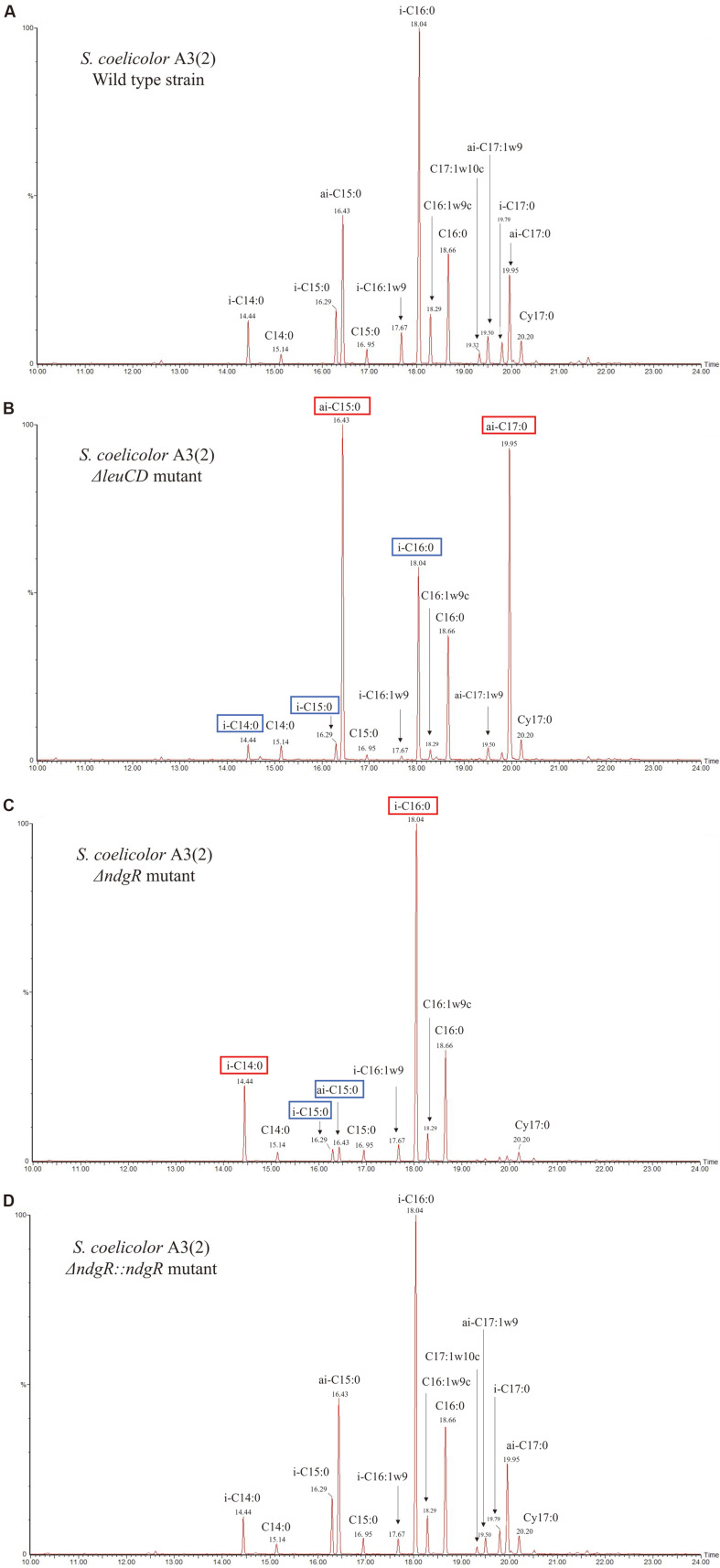
Phospholipid fatty acid peaks detected using gas chromatography-mass spectrometry (GC-MS) for the *Streptomyces coelicolor* A3(2) wild-type strain (A), Δ*leuCD* mutant (B), Δ*ndgR* mutant (C), and Δ*ndgR*::*ndgR* complementary sample (D). Fatty acids with red box mean increased in portion when compared to wild type and blue box means decreased in portion.

Cells were cultivated on R5 minimal plates covered with cellulose membrane and assessed after 48 h and 72 h, as this provided enough cells for PLFA analysis. As the PLFA analysis peaks changed with time, the GC-MS peaks at 72 h were assessed due to clear differences between the wild-type and mutant strains (Fig. 2). In the Δ*leuCD* mutant, the peaks of iso-C15 and iso-C17, which are related to leucine, were significantly decreased when compared with those of the wild type. This result indicated that leucine metabolism was not functioning properly after the deletion of *leuCD*, and that peaks of iso-C14 and iso-C16, which were related to valine, were also decreased. Interestingly, peaks of anteiso-C15 and anteiso-C17 related to isoleucine were increased. In the Δ*ndgR* mutant, as NdgR was a direct regulator of *leuCD*, similar results to the Δ*leuCD* mutant were expected; however, in the Δ*ndgR* mutant, the peak pattern was notably different to that of the wild-type strain and Δ*leuCD* mutant. Surprisingly, the peak of the anteiso-C15 greatly decreased and that of the anteiso-C17 disappeared. As NdgR was known to be involved in leucine, valine, isoleucine, methionine, and cysteine metabolism, the deletion of *ndgR* was expected to affect all BCAAs by decreasing the levels of related fatty acids, not specific fatty acids. However, the deletion of *ndgR* decreased the levels of isoleucine- and leucine-related fatty acids but increased those of valine-related fatty acids. All peaks were restored to the wild-type strain levels with *ndgR* complementation, confirming that the changes in isoleucine-related fatty acids were affected by NdgR.

### Analysis of the Compositional Changes in Mutants

**Fig. 3:**
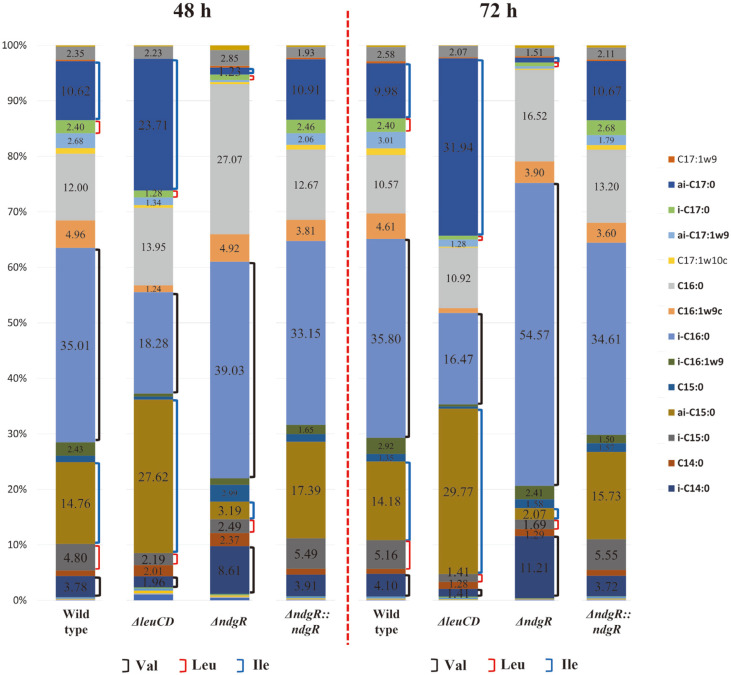
Phospholipid fatty acid composition changes in the *Streptomyces coelicolor* A3(2) wild-type strain, Δ*leuCD*, Δ*ndgR*, and Δ*ndgR*::*ndgR* after 48 h and 72 h cultivation. Branched-chain fatty acids directly related to each branched-chain amino, valine, leucine and lsoleucine are marked with black, red and blue colored line, respectively.

The compositions of the PLFAs from the wild-type strain, Δ*leuCD*, Δ*ndgR*, and Δ*ndgR*::*ndgR* mutants at 48 h and 72 h were compared (Fig. 3). The wild-type strain showed four major fatty acids [i-C_16:0_ (35.0%), ai-C_15:0_ (14.8%), C_16:0_ (12.0%), and ai-C17:0 (10.6%)] at 48 h, and there was no noticeable change in the composition at 72 h [i-C_16:0_ (35.8%), ai-C_15:0_ (14.2%), C_16:0_ (10.6%), and ai-C17:0 (10.0%)]. In Δ*leuCD*, the pattern differed from that of the wild-type strain at 48 h [i-C_16:0_ (19.3%), ai-C_15:0_ (27.6%), C_16:0_ (14.0%), and ai-C17:0 (23.7%)] and 72 h [i-C_16:0_ (16.5%), ai-C_15:0_ (30.0%), C_16:0_(10.9%), and ai-C17:0 (31.9%)]. Furthermore, the level of leucine-related fatty acids, such as i-C_15:0_ and i-C17:0, was decreased from 5.2% and 2.4% in the wild-type strain to 1.4% and 0.7% at 72 h, respectively. Meanwhile, the level of valine-related fatty acids, such as i-C_16:0_ and i-C14:0, was significantly decreased from 35.8% to 16.5% and from 4.1% to 1.4% at 72 h, respectively. Interestingly, as shown by the GC-MS peaks from the PLFA analysis, the level of isoleucine-related fatty acids, such as ai-C_15:0_ and ai-C17:0, largely increased in the Δ*leuCD* mutant from 14.2% and 10.0% to 30.0% and 31.9%, respectively, at 72 h. As LeuCD was a biosynthetic pathway enzyme like isopropylmalate dehydratase, the role as a regulator by LeuCD was not expected. Consequently, the increase in the level of isoleucine-related fatty acids seemed to be due to the decrease in both leucine- and valine-related fatty acids. This suggested that the synthesis of BCAAs and their derivatives was highly linked. In Δ*ndgR*, the change in composition was more dramatic, and the major fatty acids were modified at 48 h [i-C_16:0_ (39.3%), C_16:0_ (27.1%), C16:1w9c (4.9%) and i-C14:0 (8.6%)] and 72 h [i-C_16:0_ (54.6%), C_16:0_ (16.5%), C16:1w9c (3.9%), and i-C14:0 (11.2%)] when comparing to those of wild-type strain. The levels of the major components of the wild-type strain, such as ai-C_15:0_ and ai-C17:0, were significantly decreased to minor compositions at 72 h in Δ*ndgR* [ai-C_15:0_ (2.1%) and ai-C17:0 (>0.7%)]. Consequently, the levels of isoleucine-related fatty acids, such as ai-C_15:0_ and ai-C17:0, decreased from 14.2% and 10.0% to 2.1% and less than 0.7%. Levels of leucine-related fatty acids such as i-C_15:0_ and i-C17:0 were also decreased from 5.2% to 1.7%and 2.4% to 0.7% at 72 h, respectively. However, levels of valine-related fatty acids such as i-C_16:0_ and i-C14:0 largely increased from 35.8% to 54.6% and 4.1% to 11.2% at 72 h, respectively.

**Fig. 4:**
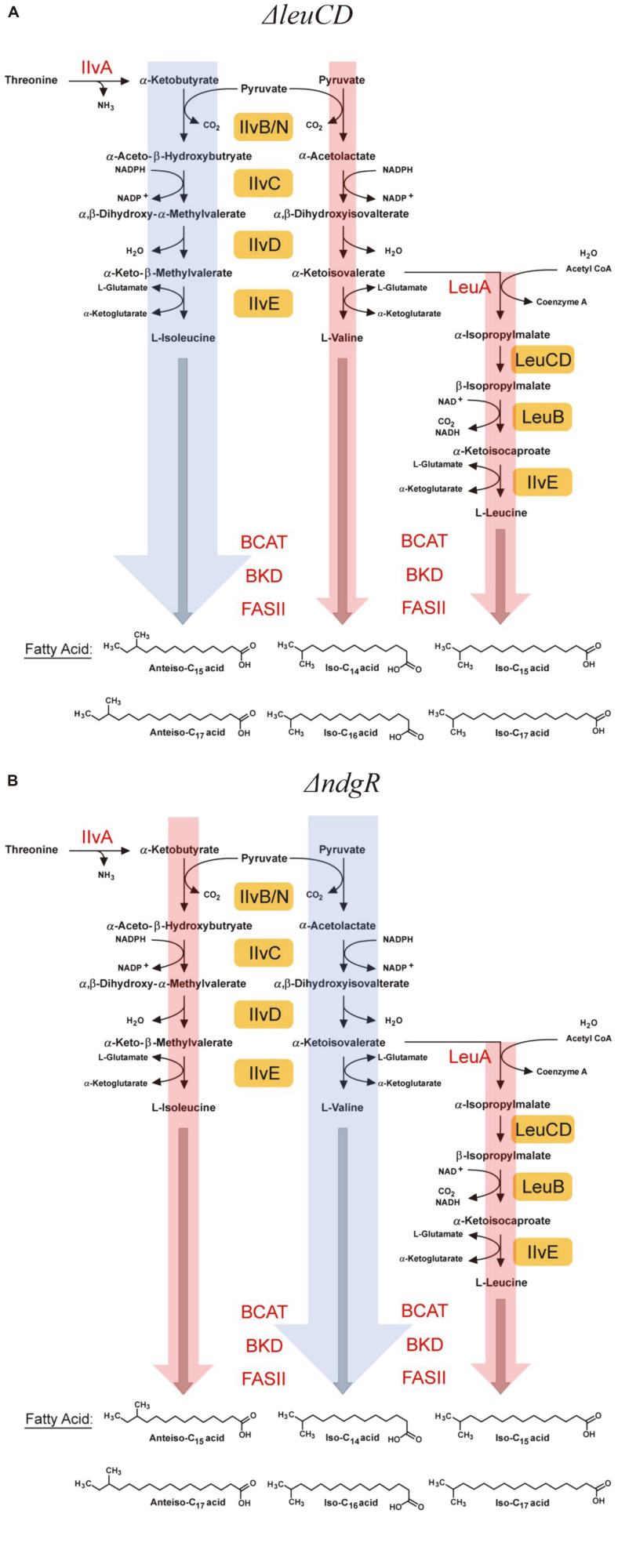
Overall changes to the fatty acid fluxes in the Δ*leuCD* (A) and Δ*ndgR* (B) mutants. The wide blue arrows indicate an increase in the fatty acid composition due to the deletion of genes and the narrow red arrows indicate a decrease in the fatty acid composition.

As NdgR governed *ilvB/N*, *ilvC*, *ilvD*, *ilvE*, *leuCD*, and *leuB* covered all BCAA biosynthesis as an activator, it was interesting that an increase in valine-related fatty acids was detected, but that there was a decrease in the levels of both isoleucine- and leucine-related fatty acids in Δ*ndgR* (Fig. 4). NdgR was a known activator of LeuCD, and there was an expected decrease in leucine synthesis with the deletion of *ndgR*; however, the decrease in isoleucine-related fatty acids and the increase in valine-related fatty acids were not easily expected. The decrease in leucine synthesis might increase the amount of valine by decreasing the bypass to leucine, or reversibly decrease valine, as with Δ*leuCD*, due to the overall decrease in the flux to leucine. However, the two different fluxes to isoleucine and valine were highly linked as they utilized the same enzymes, such as IlvB/N, IlvC, IlvD and IlvE, although isoleucine synthesis started from α-ketobutyrate and valine synthesis started from pyruvate. Different changes in isoleucine- and valine-related fatty acids were a relatively unique result and the subsequent effect on the membrane of the *ndgR* deletion was not easily explained. Although NdgR worked as an activator of *ilvB/N*, *ilvC*, *ilvD*, and *ilvE*, at the level of gene expression, the consequences could be quite different from the case of isoleucine and valine, suggesting that NdgR has a complex role in BCAA metabolism. Furthermore, it was not easy to assume that NdgR was an activator on both pathways and the level of metabolites as NdgR seemed to affect an upper region of the α-ketobutyrate and pyruvate pathway to control the flux of each precursor. Overall, the changes in flux could be summarized based on the membrane fatty acid composition, suggesting that Δ*leuCD* increased levels of isoleucine-related fatty acids and Δ*ndgR* increased those of valine-related fatty acids (Fig. 4). The complementation strain restored all reported differences in Δ*ndgR* to the levels in the wild-type strain, suggesting that the dramatic change in the BCAAs was due to the deletion of *ndgR*.

### Comparison of Growth under Cold Shock Conditions with Additional BCAAs

As there were various changes in the PLFAs in the deletion mutant Δ*ndgR*, the properties of the membrane might also change. Previously, in *Bacillus subtilis*, the deletion mutant *bkd*, which was a branched-chain alpha-keto acid dehydrogenase complex, showed decreases in ai-C_15:0_ and ai-C17:0, from 25% and 10% to 12% and 2%, respectively. Furthermore, the relative amount of the isoform of this BCAA increased, resulting in significant anteiso/iso ratio decreases, and consequently, these cells were defective in their maintenance of membrane fluidity [36]. As a result, this mutant was greatly impaired by cold shock when compared to the wild-type strain due to the change of membrane during growth at low temperatures, even in the presence of the iso-form of BCFAs [16]. Unlike *Bacillus*, in *S. avermitilis*, the deletion of *bkd* only resulted in the production of straight-chain fatty acids, and its control of membrane fluidity was dependent on unsaturated fatty acids, dramatically increasing its fluidity when compared to the wild-type strain [37].

**Fig. 5:**
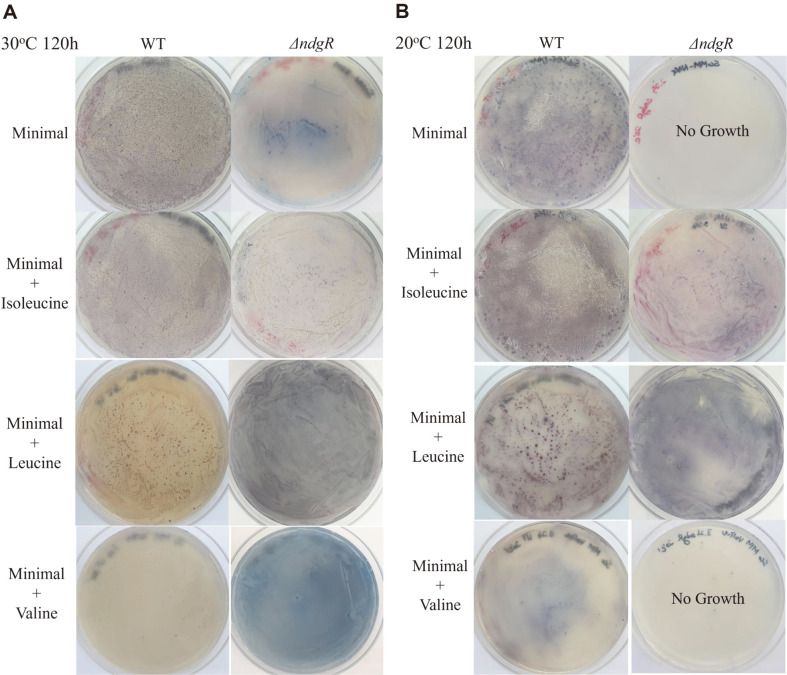
Comparison of growth at 30°C (A) and 20°C (B) with the addition of branched-chain amino acids. At 30°C, both *Streptomyces coelicolor* A3(2) wild-type strain and Δ*ndgR* mutant grew well with and without branched-chain amino acids supplementation. However, at 20°C only isoleucine and leucine supplied Δ*ndgR* mutant could grow.

As we reported the vulnerability of the Δ*ndgR* mutant to physical stress, the low growth of the Δ*ndgR* mutant was shown with shaking and the addition of glass beads to a liquid media [15]. In addition to physical stress, to test the effects of cold shock, the wild-type strain and Δ*ndgR* mutant were cultured at 20°C and 30°C. This resulted in the defective growth of the Δ*ndgR* mutant at 20°C (Fig. 5), as it could not grow in the minimal media plate, whereas the wild-type strain could grow. When 0.02% BCAAs were added to the minimal plate, the growth of the Δ*ndgR* mutant was recovered to that of the wild-type strain and it showed similar growth, except with valine supplementation, suggesting that the cold adaptation could be complemented by isoleucine and leucine in Δ*ndgR*, which was shown to be decreased in Δ*ndgR* by PLFA analysis. Supplementation of valine could not recover the growth suggesting that the mutant already has enough flux to valine, which was expected from the composition. This indicated that valine seemed less essential than isoleucine for cold shock responses in the Δ*ndgR* mutant.

The responses of both the isoleucine and leucine of *Streptomyces* were different from those reported for *Bacillus*, which showed that isoleucine was only effective in the *bkd* deletion mutant, and this data showed that NdgR worked as an activator for both the leucine and isoleucine fatty acid pathways but worked as a repressor for the valine fatty acid pathway.

Considering the importance of isoleucine in controlling the anteiso/iso ratio, which is crucial for the fluidity of bacteria, the results of this study have revealed a new link between NdgR and isoleucine and the reason for the vulnerability of the Δ*ndgR* mutant to various physical shocks.

### PLFA Analysis of the Δ*ndgR* Mutant after BCAA Complementation

**Fig. 6:**
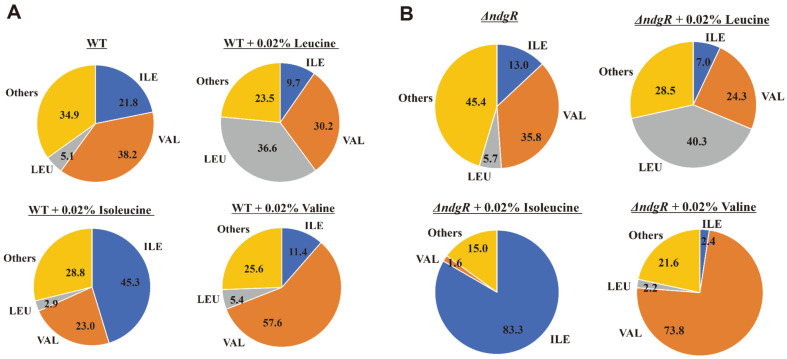
Phospholipid fatty acid analysis results for the *Streptomyces coelicolor* A3(2) wild-type strain (A) and Δ*ndgR* (B) with and without branched-chain amino acid supplement. ILE, VAL, LEU, and Others means each branched-chain related fatty acid, respectively.

After monitoring the growth recovery with the addition of isoleucine, the changes in the PLFAs with BCAA complementation were assessed (Fig. 6). The wild-type strain and Δ*ndgR* mutant with and without BCAAs were utilized. The cells were cultivated on the R5 minimal plate with or without the BCAAs, covered with a cellulose membrane, collected after 72 h, and the PLFA compositions compared.

The addition of leucine increased the levels of leucine-related fatty acids, such as iso-C14 and iso-C16, in both the wild-type strain and Δ*ndgR* mutant, while the composition of the isoleucine-related fatty acids decreased (Fig. 6A). Their PLFA patterns were notably different without the amino acid additions; however, after the addition of leucine, they were similar, suggesting that leucine metabolism was highly controlled by NdgR, as previously reported [11]. Furthermore, the addition of leucine could complement the PLFA of the Δ*ndgR* mutant and enabled cells to survive under cold shock conditions (Fig. 6B).

In contrast, the PLFA patterns after the addition of both isoleucine and valine were different. The addition of isoleucine increased ai-C15 and ai-C17 compositions in the wild-type strain, resulting in an increase in the overall composition of the anteiso form from 21.8% to 45.3%. In the Δ*ndgR* mutant, compared with the wild-type strain, the increases in ai-C15 and ai-C17 compositions were considerably different. In the media without the isoleucine supplement, 13.0% of the PLFA were isoleucine related, and when isoleucine was complemented, this increased to 83.5%. The levels of valine-related fatty acids decreased from 35.8% to 1.6% and leucine-related fatty acids were not observed as *leuCD* was regulated by NdgR. This meant that even in the Δ*ndgR* mutant, other enzymes related to fatty acid synthesis, such as *ilvB/N*, *ilvC*, *ilvD*, and *ilvE*, were functioning normally as isoleucine-related fatty acids were produced. However, in Δ*ndgR*, the supply of isoleucine was low and other genes related to the supply of isoleucine and valine seemed to be regulated by NdgR resulting in different PLFA patterns. As the Δ*ndgR* mutant showed different patterns from the those of the wild-type strain even after the addition of isoleucine, it was capable of surviving at 20°C, suggesting the survival mechanism to cold shock with isoleucine is slightly different from that with supplemented leucine. This showed that the defect in isoleucine metabolism was due to the deletion of *ndgR* and that isoleucine has crucial functions in *S. coelicolor*. Previously, the importance of an exogenous source of isoleucine for the increase in anteiso-branched fatty acids was emphasized and it was noted that the addition of isoleucine could result in cold-protective effects, restoration of the growth rate, and the same final culture density as that of the *bkd* mutant of *Bacillus* [38, 39]. Likewise, isoleucine appeared to function in *S. coelicolor*, which also utilized isoleucine.

The PLFA patterns of the Δ*ndgR* mutant with valine also differed, as the composition of valine-related fatty acids showed a greater increase, suggesting that the isoleucine/valine-related enzymes also functioned normally; however, for some unknown reason, the supply of precursors was severely inhibited.

## Discussion

Whenever a regulator was identified, it was routine to then identify the binding sites and monitor the mRNA expression levels of the target genes by comparing the wild-type strain with a deletion mutant. Transcriptomic and proteomic analyses could also be utilized to identify the targets and the effects of the deletion of a specific regulator. By monitoring the expression of important genes, the functions of regulators on specific targets could be determined. By applying these procedures, we could then identify the functions of various regulators and their regulons. However, when a regulator had many targets controlling related pathway enzymes, simple experiments evaluating the changes in mRNA expression were insufficient to explain the role of a regulator in this complex system. Consequently, simple binary classifications to define a regulator as either an activator or repressor should be used cautiously, as the regulator might function as a more or less powerful activator in different pathways. Assessments of regulator functions were thus very complex and sometimes only a degree of regulation should be suggested. In this investigation, we evaluated the complex regulation systems by which NdgR controls the amino acids leucine, valine, and isoleucine, to help identify its major targets. The PLFA analysis showed that the control of valine and isoleucine was similar to that previously reported for leucine. Furthermore, the composition ratios of the valine- and isoleucine-related fatty acids were found to be reversely correlated in the Δ*ndgR* and Δ*leuCD* mutants. Isoleucine was also found to be largely affected in the Δ*ndgR* mutant, resulting in large decreases in the levels of isoleucine-related fatty acids, and these changes seemed to affect the responses in the fluidity of the membrane and cold shock-related phenomena. Although LeuCD was previously found to be regulated by NdgR, the change in isoleucine appeared to be significant and the effects were more dramatic. The target genes of NdgR, especially the isoleucine-related genes, were known; however, the upper region of the α-ketobutyrate pathway and the isoleucine supply seemed to be more dramatically affected than valine, although valine and isoleucine shared common enzymes.

Although the exact function and importance of isoleucine has not previously been well elucidated in *Streptomyces*, the isoleucine-related fatty acids were expected to be quite important for cold shock responses in *Streptomyces*. In addition, our results also showed that the complementation of isoleucine and leucine could recover the growth defects.

**Fig. 7:**
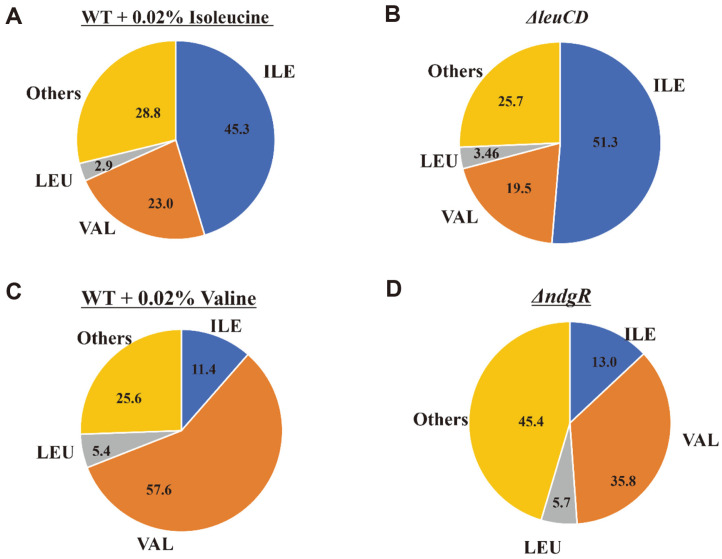
Phospholipid fatty acid analysis results for the *Streptomyces coelicolor* A3(2) wild-type strain (A) and Δ*leuCD* mutant (B) in supplemented with isoleucine and the wild-type strain (C) and Δ*ndgR* (D) supplemented with valine. ILE, VAL, LEU, and Others means each branched-chain related fatty acid, respectively.

Interestingly, as expected, there was an increased flux to isoleucine and decreased flux to valine and leucine in the Δ*leuCD* mutant, when compared with that in the Δ*leuCD* and the wild-type strain with the addition of leucine. Furthermore, they showed similar PLFA compositions, suggesting that the deletion of *leuCD* increased the isoleucine flux to isoleucine-related fatty acids, which is similar to the supply of isoleucine in the wild-type strain (Figs. 7A and 7B). For the Δ*ndgR* mutant, an increased flux to valine and decreased flux to leucine and isoleucine were expected, and the composition of the isoleucine- and leucine-related fatty acids was similar to that of the wild-type strain with the addition of valine (Figs. 7C and 7D). However, the valine-related fatty acids differed due to the different amounts of valine.

Overall, the functions of the global regulator NdgR were found to be deeply involved in BCAA synthesis, especially isoleucine and valine with different mechanisms, and the changes were clearly identified using PLFA analysis. The application of PLFA analysis in *Streptomyces* identified the direct effects on BCAA synthesis. Therefore, the findings of this study could provide insights and approaches for further investigation of *ndgR* and related regulators involved in various metabolic pathways, as revealed through fatty acid composition monitoring. Specifically, the regulation of NdgR on branched-chain amino acids to fatty acids might be extended to other amino acids to fatty acids, which could potentially lead to the control of cell viability and resistance to environmental stress for improved production of antibiotics and other drugs.
